# Spatial Distribution of Temporalis Pressure Pain Sensitivity in Men with Episodic Cluster Headache

**DOI:** 10.3390/ijerph16214239

**Published:** 2019-11-01

**Authors:** María Palacios-Ceña, Stella Fuensalida-Novo, María L Cuadrado, Carlos Ordás-Bandera, Pascal Madeleine, César Fernández-de-las-Peñas, Ángel L. Guerrero

**Affiliations:** 1 Department of Physical Therapy, Occupational Therapy, Occupational Therapy, Physical Medicine and Rehabilitation, Universidad Rey Juan Carlos, 28922 Alcorcón, Spain; 2 Center for Neuroplasticity and Pain (CNAP), Center for Sensory-Motor Interaction (SMI), Department of Health Science and Technology, Faculty of Medicine, Aalborg University, 9220 Aalborg, Denmark; 3 Department of Neurology, Hospital Clínico San Carlos, 28922 Madrid, Spain; mlcuadrado@med.ucm.es; 4 Department of Neurology, Hospital Rey Juan Carlos, 28922 Madrid, Spain; ordas.carlos@gmail.com; 5 Sport Sciences-Performance and Technology, Department of Health Science and Technology, Aalborg University, 9220 Aalborg, Denmark; pm@hst.aau.dk; 6 Headache Unit, Hospital Clínico Universitario de Valladolid, 47003 Valladolid, Spain; gueneurol@gmail.com; 7 Institute for Biomedical Research of Salamanca (IBSAL), 37007 Salamanca, Spain

**Keywords:** cluster headache, pressure pain, topographical maps, sensitization

## Abstract

(1) Background: Spatial changes in pressure sensitivity have been described in migraine and tension-type headaches. Our aim was to determine differences in the spatial distribution of pressure pain sensitivity of the temporalis muscle between cluster headache (CH) patients and headache-free controls; (2) Methods: Pressure pain thresholds (PPTs) were determined over nine points covering the temporalis muscle in 40 men with episodic CH and 40 matched headache-free controls in a blinded fashion. Topographical pressure pain sensitivity maps were constructed based on interpolation of the PPTs. Patients were evaluated in a pain-free period (remission phase), at least 3 months from the last attack and without medication; (3) Results: The analysis of covariance (ANCOVA) found significant difference between points (F = 21.887; P < 0.001) and groups (F = 24.416; P = 0.602), but not between sides (F = 0.440; P = 0.508). No effect of depression (F = 0.014; P = 0.907) or anxiety (F = 0.696; F = 0.407) was observed. A post-hoc analysis revealed: 1) lower PPTs at all points in patients than in controls, 2) an anterior-to-posterior gradient in patients but not in controls, with lower PPTs located in the anterior column. Large between-groups effects were shown in all points (standardized mean difference, SMD > 0.8); (4) Conclusions: Bilateral pressure pain hypersensitivity to pressure pain in the temporalis muscle and an anterior-to-posterior gradient to pressure pain was observed in men with episodic CH.

## 1. Introduction

Cluster headache (CH) is a primary headache disorder classified as a trigeminal autonomic cephalalgia, with an overall men-to-women preponderance (ratio 3:1) and a lifetime prevalence of 124 per 100,000 [1]. Current pathogenic theories on CH underline the role of the posterior area of the hypothalamus, the activation of the trigemino-vascular system, and the presence of an altered nociceptive pain processing [2].

Pressure pain hypersensitivity is a manifestation of altered nociceptive processing commonly observed in primary headaches, such as migraine and tension-type headache. According to a published comprehensive systematic review, pressure pain thresholds (PPTs) over temporalis, masseter, and frontalis muscles are lower in individuals suffering from migraine or tension-type headache when compared to healthy controls [3]. Of note, the temporalis muscle was found to be the most pressure-sensitive muscle [3]. There is also evidence showing the presence of pressure pain hypersensitivity over the trigeminal area in patients with CH. Yet, some authors have reported bilateral hypersensitivity to pressure pain [4,5], whereas others have described an asymmetry whereby the symptomatic side was more sensitive than the non-symptomatic side [6]. In all these studies, measurements were conducted on specific points in each of the head regions studied [4,5,6]. Bono et al. have assessed PPTs over 10 points of the headache and over the deltoid area in patients with CH. The anterior and intermediate areas of the temporal muscle on the symptomatic side were the most sensitive to pressure pain [7]. Some of the previous studies on CH included small sample sizes [4,5,6], and three were composed of both men and women with either episodic or chronic CH [5,6,7]. Additionally, factors able to elicit hyperalgesic responses within the central nervous system, such as anxiety or depression, have not been included in the analyses [4,5,6,7].

Spatial changes in sensitivity have been described in different chronic pain conditions, suggesting that the sensitivity to pressure pain is not uniformly distributed within the same anatomical region [8]. Two previous studies shaped the topographical pressure sensitivity of the temporalis muscle in patients with tension-type headache [9] and migraine [10]. Both studies revealed an anterior-to-posterior gradient of sensitivity to pressure pain in patients but not in controls, with the anterior part of the temporalis muscle being the most sensitive [9,10]. This particular spatial distribution of pressure pain sensitivity gives a potential explanation for differences observed in previous studies analyzing sensitivity to pressure pain in individuals with headache and healthy controls. We considered that topographical pressure pain sensitivity mapping of the temporalis area might also contribute to a better understanding of nociceptive pain processing in CH.

Our aim was to evaluate differences in the spatial distribution of sensitivity to pressure pain of the temporalis muscle between patients with episodic CH and matched headache-free controls by adjusting any potential effect of anxiety and depression. Our hypothesis was that individuals with CH would exhibit higher pressure pain sensitivity than healthy controls and that spatial distribution of pressure pain sensitivity would reveal an anterior-to-posterior gradient in agreement with findings from patients with tension-type headache and migraine.

## 2. Materials and Methods

### 2.1. Participants

The current paper is part of a multicenter headache study. Some of the patients of the current study were also included in a previous study [11]. This study presents new additional data and different statistical analyses.

Male patients followed for CH in two headache units were recruited between July 2018 and March 2019 and screened for eligible inclusion criteria. For patients to be eligible, they had to meet the International Classification of Headache Disorders, 3rd edition (ICHD-3) diagnostic criteria for episodic CH [12].Exclusion criteria included (1) women, (2) younger than 18 or older than 65 years, (3) headaches with side shifts, (4) diagnosis of another primary or secondary headache; (5) peripheral neuropathy or another neurological disease; (6) medical systemic disease (e.g., systemic lupus erythematosus or rheumatoid arthritis); (7) previous head or neck trauma (e.g., whiplash) or (8) previous head or neck surgery. Additionally, age-matched asymptomatic men without history of headache and without pain symptoms within the last 6 months were recruited from volunteers who responded to local announcements.

Participants read and signed a written consent form prior to their inclusion in the study. The study design was approved by local Ethic Committees of Hospital Clínico San Carlos of Madrid (code 17/513-E) and Hospital Clínico Universitario of Valladolid (code PI 17-875).

### 2.2. Clinical Data

A structured questionnaire was used to obtain demographic and clinical data, including [11] age, time since the onset of CH, number of clusters/year, time since the last cluster period, painful side in the last cluster attack and in previous attacks, intensity and duration of attacks, medication intake for attacks, and time without medication. All participants reported normal neurologic and ophthalmologic examinations as well as normal brain magnetic resonance imaging (MRI).

The Hospital Anxiety and Depression Scale (HADS) was used to determine the anxiety and depressive levels [13].In this questionnaire, seven items evaluate anxiety levels (HADS-A) and seven items evaluate depressive levels (HADS-D). The response to each item is provided in a Likert-type scale (0–3). The total sum of all answers is transformed into a global score (0–21) for each scale, where higher scores indicate higher anxiety and depressive levels [14]. The HADS has shown good validity and internal consistency in headache patients [15].

### 2.3. Pressure Pain Thresholds

An electronic pressure algometer (Somedic^®^ Algometer type 2, Rødby, Sweden) was used to determine the PPT, i.e., the amount of pressure where the sensation of pressure first changes to pain. The subjects were instructed to press the “stop-button” of the algometer as soon as the pressure resulted in the first sensation of pain. Pressure was increased at a rate of approximately 30 kPa/s with a 1 cm^2^ probe. The PPTs were assessed three times on each point, with a 30 sec resting period for avoiding temporal summation of pain [16]. The mean of the three trials was calculated and used for statistical analyses. The order of assessment was randomized among participants and the assessor was blinded to the subject’s condition. Previous studies have reported high reliability of pressure algometry [17,18].

Participants attended a preliminary session for familiarization with pressure test procedures where PPT assessment was first made on the wrist extensor muscles of the right forearm. The assessments were held in an asymptomatic and remission phase, e.g., when no headache attack had occurred for at least 3 months to ensure that the patient was not switching to a chronic form of CH [11,12] and to avoid pain-related allodynia. Additionally, preventive medication or abortive drugs had to be stopped at least 1 month before PPT assessments. No analgesic or muscle relaxation drugs were allowed at least 48 hours before testing.

### 2.4. Spatial Distribution of Pressure Pain Sensitivity

**Figure 1:**
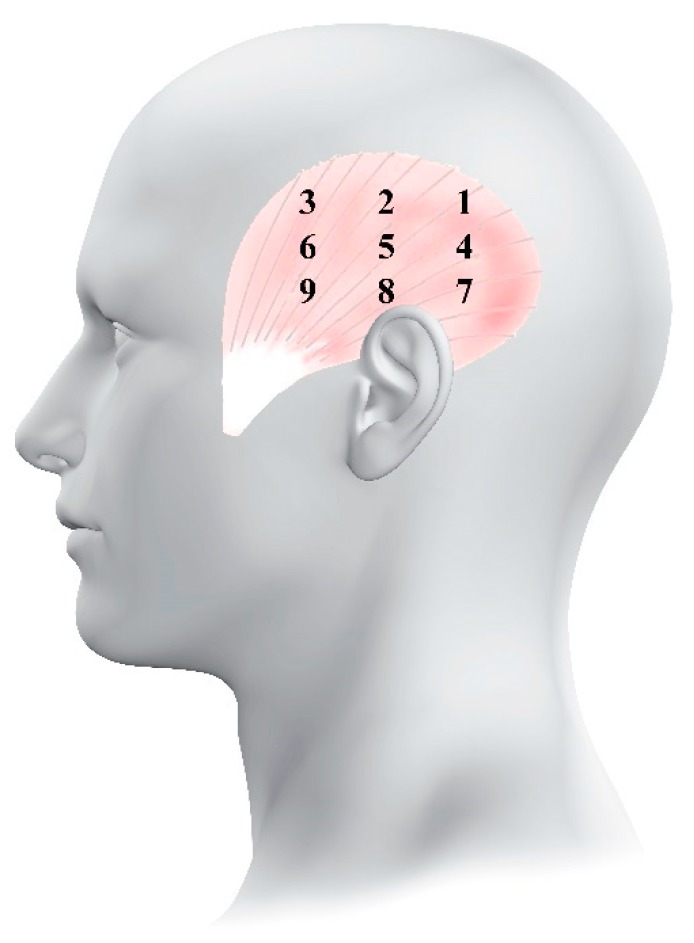
Location of the points for pressure pain threshold measurements over the temporalis muscle.

PPTs were determined in nine points over the temporalis muscle with the same protocol of previous studies [8,9,10]. The center of the temporalis muscle belly was defined with a vertical line through the ear. This line was the central column of the topographical map. Three points separated by 1.5 cm were marked over this line and denominated as 2, 5, and 8 (from top to bottom). The points located in the posterior part of the temporalis muscle were localized 1 cm posterior the central points and were labeled as 1, 4 and 7. Finally, points anatomically located in the anterior part of the temporalis muscle were located 1 cm anterior to the central points and were labeled as 3, 6 and 9 (Figure 1). With these points, the entire temporalis muscle (posterior, middle and anterior) was covered.

The average PPT values of the nine points were used to create the topographical pressure pain sensitivity maps of the CH patients and headache-free controls. For that purpose, an inverse distance weighted interpolation was used to get a graphical representation of spatial pressure pain distribution of the entire surface of the temporalis muscle. Yet, the PPT values of non-assessed locations were interpolated [19]. See the review by Alburquerque-Sendín et al. [8] for more details about mathematical model for mapping.

### 2.5. Sample Size Calculation

As previously described [11], sample size was calculated for detecting a moderate-large effect size (0.70) between patients and controls on PPT, with a two-tailed test, an alpha level (α) of 0.05 and a desired power (β) of 90%. These data generated a sample size of at least 35 participants per group. Sample size calculations were conducted with software Tamaño de la Muestra1.1© (Barcelona, Spain)

### 2.6. Statistical Analysis

Data analysis was conducted with the SPSS statistical package (22.0 Version). All demographic data are expressed as means and standard deviation, whereas PPT scores are expressed as means and 95% confidence intervals (95%CI).

Differences in PPT scores between CH patients and headache-free controls were calculated with a mixed-model analysis of covariance (ANCOVA) with the PPT point (1 to 9) and side (symptomatic/non-symptomatic in CH patients, dominant/non-dominant within headache-free controls) as the within-subjects factors, group (episodic CH/ headache-free controls) as between-subjects factor and depressive/anxiety level scores as covariates. When statistically significant differences were observed, the Bonferroni test was used as post-hoc comparisons. For multiple comparisons conducted during mixed-model ANCOVA, a Bon-ferroni-corrected alpha level of 0.006 (9 points of assessment) was considered statistically significant. Between-groups clinical significances for PPTs were determined by calculating standardized mean differences (SMD), e.g., dividing the between-group difference by the pooled standard deviation. For SMD, values ranging from 0.0 to 0.2 were considered trivial, those ranging from 0.2 to 0.49 were considered small, moderate when ranging from 0.5 to 0.79, and large when greater than 0.8. Finally, the potential association between headache pain variables and PPT scores were analyzed with the non-parametric Spearman’s rho (r_s_) test.

## 3. Results

### 3.1. Clinical Features of the Sample

Fifty individuals suffering from CH were screened for potential eligibility. Ten (20%) patients presented some exclusion criteria: chronic CH (n = 4), concomitant diagnosis of migraine (n = 3) and during an active cluster period (n = 3). Finally, 40 men with episodic CH (mean age, 42; SD, 5) and 40 matched headache-free men serving as controls (mean age, 41; SD, 4) were included. All patients had unilateral CH with no side shifts (19 left side, 21 right side). Table 1 summarizes clinical data of both groups [11].

### 3.2. Spatial Distribution of Pressure Pain Sensitivity

The results revealed significant between-point (F = 21.887; P < 0.001) and between-group (F = 24.416; P = 0.602), but not between-sides (F = 0.440; P = 0.508) differences for PPTs. The post-hoc analysis revealed significantly lower PPTs (P < 0.001) in all the points in men with episodic CH compared with headache-free controls (Table 2). No significant effects of depressive (F = 0.014; P = 0.907) or anxiety (F = 0.696; F = 0.407) levels were seen.

**Figure 2:**
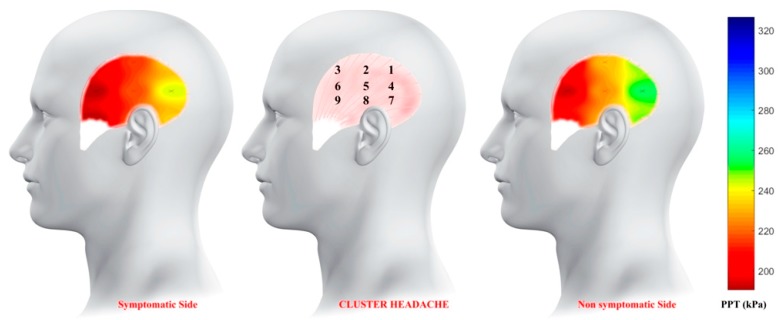
Topographical maps showing spatial distribution of pressure pain sensitivity of the temporalis muscle in men with cluster headache. “X” marks the location of the points where the pressure pain threshold was measured.

**Figure 3:**
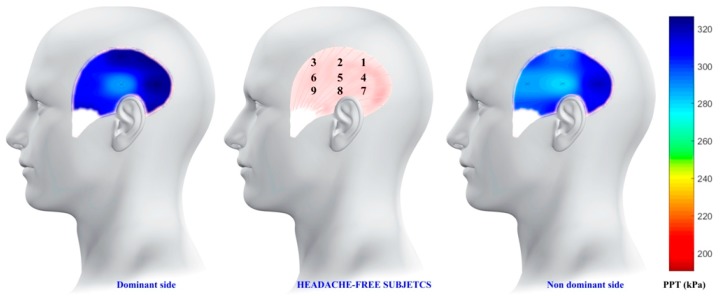
Topographical maps showing spatial distribution of pressure pain sensitivity of the temporalis muscle in headache-free subjects. “X” marks the location of the points where the pressure pain threshold was measured.

A significant Group * Points (F = 9.046; P < 0.001), but not Group * Side * Points (F = 1.242; P = 0.284), interaction was observed (Table 2). In men with episodic CH, PPTs showed a bilateral anterior-to-posterior gradient of pressure pain with the most sensitive points located in the anterior part of the temporalis muscle (points 3, 6, 9), followed by those within the central part (points 2, 5, 8) and those in the posterior part (points 1, 4, 7) (P < 0.001, Figure 2). Within headache-free controls, PPTs did not follow such a gradient, i.e., the most sensitive point was the mid-muscle belly (point 5, Figure 3).

### 3.3. Inter-Measure Comparisons of Effect Size

In general, large effects were observed between men with episodic CH and headache-free controls comparisons for PPTs for all points (all, SMD > 0.80). In fact, effect sizes were larger for the anterior part (SMD point 3, 6 and 9:1.70, 1.60 and 1.60, respectively) compared with the posterior (SMD point 1, 4 and 7:1.07, 1.07 and 0.95) and central (SMD point 2, 5 and 8:1.20, 0.8 and 1.05, respectively) parts. Nevertheless, it should be observed that the lowest effect size was found to be > 0.50, probably due to the heterogeneous distribution of the maps within headache-free controls.

### 3.4. Associations with Headache Features

Topographical pressure sensitivity maps were not significantly associated with any of the headache pain features (all, P > 0.180).

## 4. Discussion

We found bilateral hypersensitivity to pressure pain within the temporalis muscle in men with episodic CH not to be associated with levels of anxiety or depression. Additionally, our mapping revealed a spatial anterior-to-posterior gradient of sensitivity to pressure pain in men with CH but not in headache-free controls.

In line with our hypothesis, there was a bilateral decrease of PPTs in men with CH compared with controls with large effect sizes. Our results agree with those previously reported [4,5] where bilateral hypersensitivity to pain pressure in the temporalis area in patients with CH was also observed. However, these results differ from those reported by Malo-Urriés et al. [6], who observed that the symptomatic side was more sensitive than the non-symptomatic side in a small sample of patients with CH. The current and previous findings suggest the presence of sensitization of trigeminal pain pathways in CH patients. In addition, the presence of bilateral pressure pain hyperalgesia in patients with unilateral symptoms supports potential sensitization of the central nervous system, as previously suggested [2], although further studies are needed. Similarly, bilateral hyperalgesia to pressure over the temporalis muscle has previously been found in patients with strictly unilateral migraine [10], suggesting that bilateral hypersensitivity is present albeit the presence of just unilateral symptoms in individuals with primary headaches.

Our patients were assessed during a long-lasting remission phase. Therefore, pressure pain hyperalgesia in the trigeminal area seems to be present in patients with CH during asymptomatic periods. These findings indicate that there is a baseline state of trigeminal sensitization in CH predisposing to periods of pain. Alternatively, it is also possible that the altered nociceptive processing could be induced by repetitive headache attacks of high intensity and then maintained without the presence of any nociceptive stimuli during the remission phase. Indeed, the occurrence of long-term potentiation induced by nociceptive stimuli has been suggested in other primary headaches [20,21]. However, we cannot confirm the presence of central sensitization in CH, since PPTs represent a static outcome and dynamic measures of nociception would be needed.

Pressure pain sensitivity mapping based on multi-site recordings has previously been documented as a useful technique for visualizing the spatial topographic distribution of pain sensitivity [8]. This study is the first attempt at assessing topographical pressure pain sensitivity maps in patients with episodic CH. According to our findings, in CH patients, there is an anterior-to-posterior gradient of sensitivity within the temporalis muscle so that the lowest pain thresholds are located in the anterior region of the muscle. This gradient is not present in headache-free controls, where the most sensitive area was located in the mid-muscle belly. The results of this study are similar to those previously described in other primary headaches, such as tension-type headache and unilateral migraine [9,10]. To determine the mechanisms underlying the anterior-to-posterior gradient is beyond the scope of the current paper. However, two hypotheses can be stated. First, sensitization of trigeminal pathways could generate a pressure pain hyperalgesic pattern with an anterior-to-posterior distribution in primary headaches, regardless of unilateral or bilateral location of the symptoms or the specific type of headache. Secondly, an anterior-to-posterior gradient could be also related to different distribution of muscle nociceptors between the different areas of the temporalis muscle that could predispose to headaches, but this cannot be confirmed until histological studies are conducted.

Additionally, we also found that the levels of anxiety and depression do not seem to have a significant influence on pressure pain hyperalgesia within the trigeminal area in men with episodic CH. It would be interesting to investigate other potential psychological factors related to the observed anterior-to-posterior distribution of sensitivity to pressure pain in primary headaches. In fact, differences in the spatial distribution of sensitivity to pressure pain should be considered in future studies comparing headache populations and headache-free controls, since taking the same point as a reference could be biased. In addition, changes in the spatial distribution of pressure pain within the trigeminal area could be used to monitor the effects of treatment in individuals with CH.

We must recognize some limitations of our study. First, our sample was only comprised of men with episodic CH in a remission phase. Therefore, we cannot draw conclusions concerning women with CH, or men with chronic CH, or with episodic CH in an active phase. Secondly, depressive and anxiety levels of our sample were relatively low, thus, their potential role in spatial distribution of pressure pain sensitivity should be considered with caution at this stage. Thirdly, we used a cross-sectional design, which does not permit to determine a cause and effect relationship between spatial distribution of pressure pain sensitivity and the development or maintenance of CH. However, the fact that headache-free control subjects showed heterogeneous pain maps suggests that the anterior-to-posterior gradient observed in men with CH is related to the presence of pain. Longitudinal studies are needed to clarify this hypothesis. Finally, we only assessed static outcomes of nociception, i.e., PPT. Thus, dynamic outcomes such as temporal summation, wind-up, or conditioned pain modulation should be used in future studies to further characterize the presence of central sensitization in CH.

## 5. Conclusions

We found bilateral hypersensitivity to pressure pain over the temporalis muscle in men with episodic CH in a remission (asymptomatic phase). Moreover, topographical maps revealed a spatial anterior-to-posterior gradient of pressure pain in patients with CH but not in headache-free healthy controls. This topographical pattern resembles the one already described in migraine and tension-type headache and could be a consequence of trigeminal pain pathways sensitization.

Key Findings

1 This study found bilateral hypersensitivity to pressure pain over the temporalis muscle in men with episodic CH in a remission phase.
2 The topographical pressure pain sensitivity maps of the temporalis muscle showed an anterior-to-posterior gradient in patients with CH but not in controls.
3 The spatial distribution of pressure pain in cluster headache resembles the one already described in migraine and tension-type headache.

## Figures and Tables

**Table 1:** Demographic and clinical variables of men with episodic cluster headache and healthy controls (mean ± SD).

	Cluster Headache (n = 40)	Healthy Controls (n = 40)
**Age (years)**	42.0 ± 5.0	41.0 ± 4.0
**Onset of headache attacks (years)**	13.0 ± 5.0	-----
**Cluster periods per year**	2.0 ± 1.5	-----
**Duration of the cluster period (months)**	1.7 ± 1.0	-----
**Number of attacks per day during cluster period**	2.0 ± 1.7	-----
**Time from the last cluster period (months)**	9.9 ± 3.2	-----
**Time without taking medication (months)**	9.0 ± 2.9	-----
**HADS-D (0-21) ^#^**	2.6 ± 1.1	1.2 ± 0.8
**HADS-A (0-21) ^#^**	6.3 ± 2.1	3.4 ± 1.1

^#^HADS: Hospital Anxiety and Depression Scale (D: Depression; A: anxiety). ^#^Significant differences between patients and controls (ANCOVA test, P<0.001).

**Table 2:** Pressure Pain Thresholds (PPT, kPa) of each point in men with episodic cluster headache and headache-free controls (mean (95% CI))**.**

	Cluster Headache (n = 40)*		Headache-free Controls (n = 40)	
**Point 1**	**Symptomatic Side**	234.5 (201.0, 268.0)	**Non-dominant Side**	315.5 (298.0, 333.0)
	**Non-Symptomatic Side**	250.5 (218.0, 283.0)	**Dominant Side**	326.5 (312.5, 340.5)
**Point 2**	**Symptomatic Side**	215.0 (181.0, 249.0)	**Non-dominant Side**	292.0 (278.0, 306.0)
	**Non-Symptomatic Side**	230.5 (192.0, 269.0)	**Dominant Side**	322.5 (310.0, 335.0)
**Point 3^#^**	**Symptomatic Side**	182.5 (156.5, 208.5)	**Non-dominant Side**	304.0 (290.0, 318.0)
	**Non-Symptomatic Side**	194.0 (163.5, 224.5)	**Dominant Side**	304.5 (291.0, 318.0)
**Point 4**	**Symptomatic Side**	234.5 (201.0, 268.0)	**Non-dominant Side**	316.0 (301.0, 331.0)
	**Non-Symptomatic Side**	255.5 (217.5, 293.5)	**Dominant Side**	325.0 (311.0, 339.0)
**Point 5**	**Symptomatic Side**	216.5 (186.0, 247.0)	**Non-dominant Side**	280.5 (264.5, 296.5)
	**Non-Symptomatic Side**	226.5 (191.0, 262.0)	**Dominant Side**	288.0 (270.0, 306.0)
**Point 6^#^**	**Symptomatic Side**	181.5 (156.0, 207.0)	**Non-dominant Side**	287.0 (272.0, 302.0)
	**Non-Symptomatic Side**	193.5 (162.0, 225.0)	**Dominant Side**	307.0 (292.0, 322.0)
**Point 7**	**Symptomatic Side**	234.0 (201.0, 267.0)	**Non-dominant Side**	313.0 (295.0, 331.0)
	**Non-Symptomatic Side**	249.5 (210.5, 288.5)	**Dominant Side**	324.0 (308.5, 339.5)
**Point 8**	**Symptomatic Side**	216.0 (183.0, 249.0)	**Non-dominant Side**	309.0 (293.0, 325.0)
	**Non-Symptomatic Side**	229.0 (189.0, 269.0)	**Dominant Side**	304.0 (287.0, 321.0)
**Point 9^#^**	**Symptomatic Side**	191.0 (165.0, 217.0)	**Non-dominant Side**	299.0 (284.0, 314.0)
	**Non-Symptomatic Side**	207.0 (174.0, 240.0)	**Dominant Side**	316.0 (302.5, 339.5)

**^#^** Significant differences between points 3, 6, and 9 and the remaining points; * Significant difference between groups (ANCOVA, P < 0.001).
